# Isolation and Characterization of Melanoidins from Dulce de Leche, A Confectionary Dairy Product

**DOI:** 10.3390/molecules24224163

**Published:** 2019-11-17

**Authors:** Analía Rodríguez, Patricia Lema, María Inés Bessio, Guillermo Moyna, Luis Alberto Panizzolo, Fernando Ferreira

**Affiliations:** 1 Departamento de Ciencia y Tecnología de Alimentos, Facultad de Química, Universidad de la República, General Flores 2124, Montevideo C.P. 11800, Uruguay; analiarb@gmail.com (A.R.); apanizzo@fq.edu.uy (L.A.P.); 2 Instituto de Ingeniería Química, Facultad de Ingeniería, Universidad de la República, Julio Herrera y Reissig 565, Montevideo C.P. 11300, Uruguay; plema@fing.edu.uy; 3 Laboratorio de Carbohidratos y Glicoconjugados, Departamento de Química Orgánica, Facultad de Químicay Departamento de Desarrollo Biotecnológico, Facultad de Medicina, Universidad de la República, Dr. Alfredo Navarro 3051, Montevideo C.P. 11600, Uruguay; mibessio@fq.edu.uy; 4 Departamento de Química del Litoral, CENUR Litoral Norte, Universidad de la República, Ruta 3 Km 363, Paysandú C.P. 60000, Uruguay; gmoyna@fq.edu.uy

**Keywords:** color, dulce de leche, maillard, melanoidins, milk

## Abstract

Melanoidins, the brown-colored compounds formed through the Maillard reaction, are responsible for color development in dulce de leche (DL), a popular confectionary dairy product in the Río de la Plata region, particularly in Uruguay and Argentina. Color is a critical quality parameter that strongly influences consumer preference. This work aimed to develop a method to perform preliminary structural characterization of the chromophores produced by the Maillard reaction. Melanoidins are present in a water-insoluble fraction, linked to a protein backbone, conforming melanoproteins of high molecular weight. The insoluble melanoprotein fraction (10% total solids) was isolated, and the chromophores released by proteolysis and isolated by gel-permeation chromatography. The analysis of the products revealed that they present a high degree of molecular weight (MW) polydispersity, in a range of 300 to 2000 Da, where the compounds of higher molecular weight contributed the most to the color of the product. The isolated fractions were further analyzed by RP-HPLC using a diode array detector (DAD) detector. These results, together with H-NMR data, suggested that the chromophores isolated belonged to a relatively simple mixture of aromatic products with higher hydrophobic character relative to other products of the melanoprotein digestion.

## 1. Introduction

The melanoidins are a greatly diverse group of colored compounds formed by the Maillard reaction between sugars and proteins. The study of melanoidins in food products has been a subject of increasing interest, with reports multiplying particularly in recent years, notwithstanding the inherent difficulties associated with their intricate mechanisms of formation and the great diversity of resulting products.

Most studies have focused mainly on the degree of advance of the reaction, the generation of colored compounds, and the different reaction mechanisms that led to their formation. In many foodstuffs, their potential biological properties have been studied, including genotoxicity and cytotoxicity, antioxidant activity, antimicrobial activity, and as probiotics and antihypertensives [1]. These compounds are also capable of different binding ligands and aromatic compounds [2].

The biological and technological properties of melanoidins explain the need for detailed and comprehensive knowledge of their chemical structure. In particular, color is a sensory characteristic crucial to defining consumer acceptability for many food products, which makes relevant the study and control of color development during food processing.

While the local name can vary, dulce de leche (DL) is a popular dairy product in many South American countries. It was initially a homemade product but had a rapid industrial development, and presently it is a very popular and widely accepted commercial product. This confectionery product is produced by the concentration of cow’s milk and sugar by heating. It presents characteristic color, flavor, and texture due to the extensive nonenzymatic browning that occurs during the process [3]. Color is an essential characteristic of the product because it strongly influences consumer’s preferences [4].

In this regard, DL composition is a relevant research subject since the melanoidins formed during production play an important role in color formation. Color development and Maillard Reaction Products (MRPs) have been extensively studied in concentrated milk systems [5], but there is no information at all regarding of melanoidins as the colored compound formed by the Maillard Reaction (MR) in DL.

The study of melanoidins requires their isolation from different food products, and complex matrices were these compounds are linked to other components like polyphenols [6] or proteins [7,8]. Frequently, a process to free the chemically bound melanoidins from the matrix is required, and depending upon their structure, and molecular weight, a combination of chromatographic techniques is employed for the isolation of the products. Traditionally, paper or thin layer chromatography and low or high-pressure liquid chromatography are employed for low molecular-weight (MW) melanoidins (MW < 500 Da), while dialysis, membrane filtration, ion exchange, or size exclusion chromatography are used for high molecular weight compounds [9].

In the present work, we report a methodology for the isolation and preliminary characterization of melanoidins present in DL using a combination of enzymatic, chromatographic, and spectroscopic methods.

## 2. Results and Discussion

### 2.1. Evaluation of the Insoluble Colored Fraction

The DL was prepared from cow milk and sucrose using standard industrial techniques and presented the typical characteristics of the product, in particular, a dark brown color.

**Figure 1:**
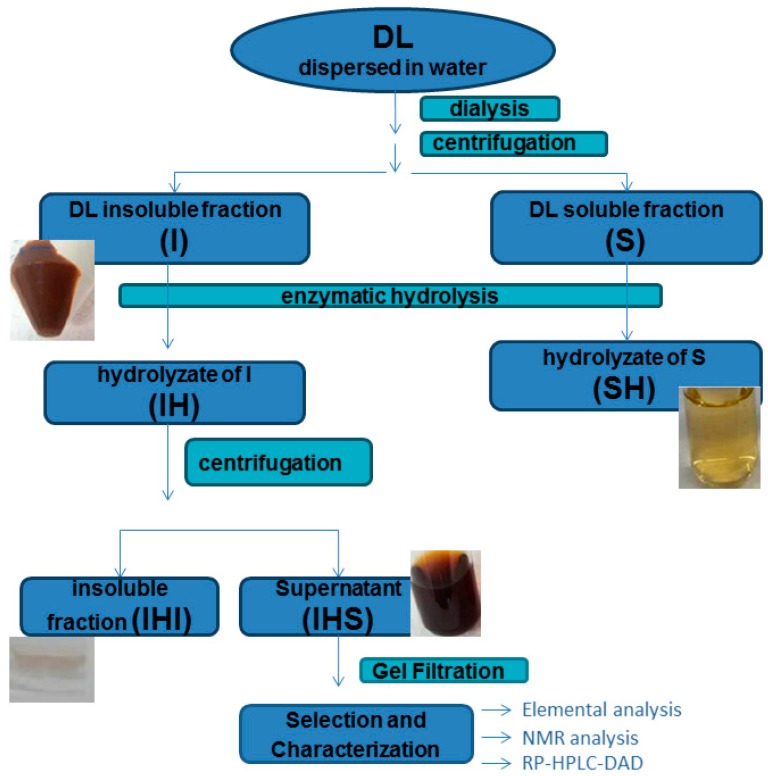
Diagram of the fractionation and isolation of different components from dulce de leche (DL), including the product after enzymatic hydrolysis of the DL soluble fraction (SH), the DL insoluble fraction (I), the soluble fraction after enzymatic hydrolysis of I (IHS), and the insoluble fraction after enzymatic hydrolysis of I (IHI).

Dispersion of DL in water yielded water-soluble (S) and water-insoluble (I) fractions, both colored, that were separated by centrifugation (Figure 1). The water-dispersion and centrifugation of the sample were repeated before and after extensive dialysis of the DL sample against water to determine whether the presence of salts and other low molecular compounds would affect the solubility and aggregation of proteins.

Table 1 shows the proportion of the S and I fractions in DL before and after dialysis. The percentage of the I fraction in DL previous to dialysis represents only 10% of the total solids, while after dialysis this value rises to approximately 30% in dry weight (Table 1). This result was expected because dialysis removes the low molecular weight components of DL, mainly lactose, sucrose, and salts.

To further characterize the insoluble fraction, it was treated enzymatically with a protease (Pronase), and the hydrolysis suspension was again fractioned by centrifugation into soluble (IHS) and insoluble (IHI) fractions. The enzymatic treatment showed almost complete hydrolysis of the insoluble fraction, supporting the idea that it was mainly an insoluble protein complex, probably formed by cross-linking between milk proteins by either intra- or inter-catenary linkages [10]. The insoluble residue after the enzymatic hydrolysis (IHI) was a lightly colored solid with a white to gray hue (Figure 1). As was previously proposed by Clark and Tannenbaum [11], this material probably consists of a mixture of peptides.

The colors of the S and I fractions were quite different, being the second more colored than the first (Figure 1). After hydrolysis of the I fraction, the color remained in the resulting soluble fraction (IHS, Figure 1). These observations suggest that the main component of the DL melanoidins is associated with the proteinaceous insoluble material forming melanoproteins.

### 2.2. Molecular Size Soluble of Melanoidins and Melanoproteins

**Figure 2:**
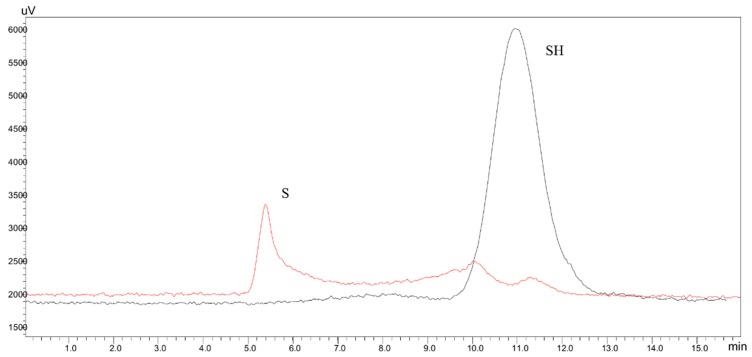
SEC-HPLC chromatograms of the soluble fraction of DL (S) and its hydrolysis products (SH).

The molecular weights of melanoidins, before and after treatment with pronase, were estimated by Size Exclusion Chromatography-HPLC (SEC-HPLC) using an SEC column with a fractionation range from 5 kDa to 100 kDa and UV detection at 280 nm (Figure 2).

The S fraction of DL showed the presence of high MW colored proteins eluting at the column void volume. Meanwhile, the SEC analysis of the hydrolysis products of this fraction (SH fraction) showed only the presence of low MW UV-absorbing compounds, as shown in Figure 2. These results indicated that the chromophoric groups present in the S fraction were originally linked to proteins. This observation is in agreement with the data reported by Hofmann [7,8] in casein-sucrose model systems where the color is due to low MW chromophores linked to proteins, giving place to high MW colored compounds.

**Figure 3:**
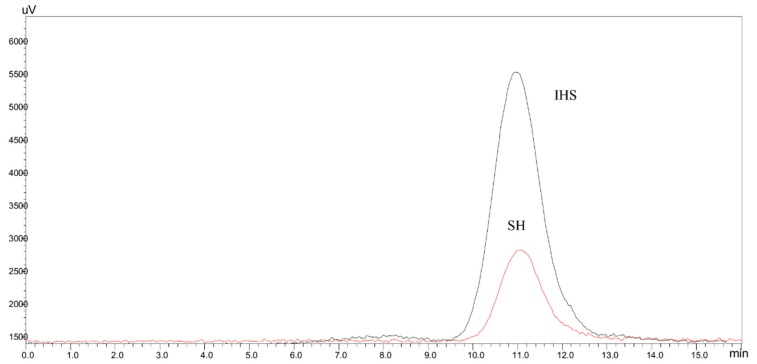
SEC-HPLC chromatograms of the hydrolysis products of the soluble (SH) and insoluble (HIS) fractions of DL.

The SEC-HPLC analysis of the hydrolysis products of the DL insoluble fraction (IHS) showed the presence of low MW colored compounds that eluted at about the same exclusion volume as the ones of SH (Figure 3), indicating that the DL colored insoluble fraction is mainly composed of protein material linked to melanoidins, released in a soluble form after proteolysis of the insoluble material. Both soluble colored fractions, released by proteolysis from the soluble and insoluble components of DL, are low-MW compounds (MW < 14.3 kDa).

### 2.3. Evaluation of Free and Bounded Colored Compounds

The amount of free and protein-bound colored products was determined, according to Morales and van Boekel [11]. Most of the colored products are bound to proteins (98.5%); just a residual amount is present in a free form (1.5%). These results are in accordance with those obtained by SEC-HPLC and findings by other authors [11,12,13].

### 2.4. Isolation of Melanoidins by Gel Filtration

**Figure 4:**
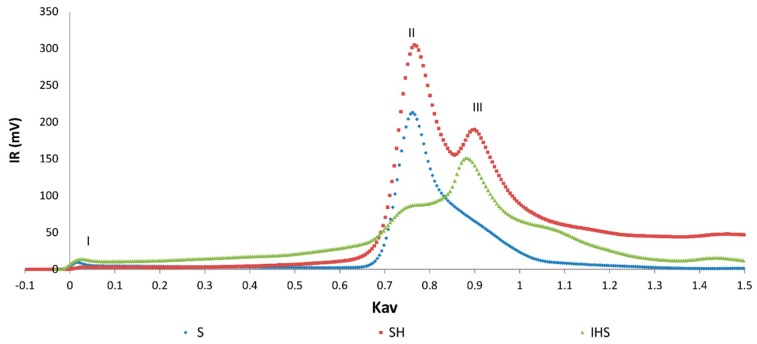
Gel filtration chromatograms on a Biogel P-2 column using IR detection of the soluble fraction of DL (S), its hydrolysis products (SH), and the hydrolysis products of the insoluble fraction (IHS). Kav: Analyte partition coefficient.

The soluble melanoidins released by enzymatic hydrolysis of the soluble and insoluble fractions of DL were isolated according to their MW by gel filtration, using refractive index (RI) detection (Figure 4). Since the increase in the refractive index of the mobile phase is proportional to the analyte concentration irrespective of its chemical nature, the relative amounts of the different fractions isolated by gel filtration may be estimated from the area of their respective peaks in the chromatogram.

The three DL soluble melanoidins samples S, SH, and IHS, showed a high size dispersion (Figure 4), including compounds eluting with the dead volume (Kav = 0) corresponding to a nominal MW ≥ 1800 Da (peak I), to low molecular components with elution volumes similar to those of monosaccharides or aminoacids.

Most of the melanoidins eluted at Kav = 0.75 Da (peak II), and in a broad peak eluting with Kav = 0.85–0.90 Peak (III), corresponding to a nominal MW of 240 and 110–142 Da, respectively, in the hydrolysis products SH and IHS. Compounds eluting the latter, with a Kav = 1, may be due to nonspecific interactions with the stationary phase [14].

**Figure 5:**
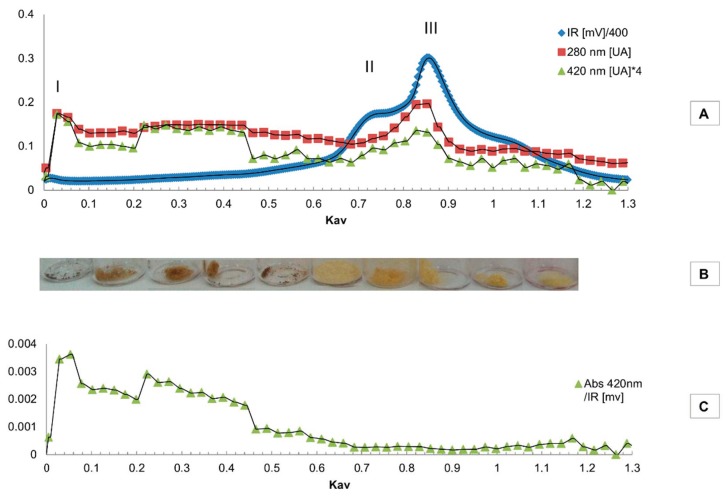
Size exclusion chromatography of IHS. IR and UV-VIS detection (**A**) and absorbance (λ 420 nm) to IR signal ratio (**C**) chromatograms. The image of the fractions is shown in (**B**). Pooled fractions mentioned in the text are indicated as I, II, and III.

The color of the freeze-dried fractions varied from almost black (peak I) to brown or beige colors (peaks II and III) (Figure 5).

The chromatograms of the HIS sample, with detection at 280 and 420 nm, show a similar pattern (Figure 5A). The material eluting in the exclusion volume represents a minor proportion of the eluting fractions, as evidenced in the RI chromatogram. However, this fraction of melanoidins are the main contributors to the sample color, as can be seen in the chromatogram with detection at 420 nm. This observation is supported by the chromatogram presented in Figure 5C, where the absorbance at 420 nm to RI ratio can be regarded as the specific absorbance of the eluting compounds, being the RI signal independent of the chemical nature of the compounds and directly related to their mass concentration in the mobile phase. As seen in Figure 5C, the compounds that contribute the most to the color of IHS eluted in the exclusion volume, pointing to a nominal MW ≥ 1800 Da. These also contribute to the lower luminosity or dark color of the IHS sample (Figure 5B), the color of the original DL, its insoluble fraction (I), and its hydrolysis product (IHS) (Figure 1).

### 2.5. Evaluation of Isolated Melanoidins by UV-VIS

**Figure 6:**
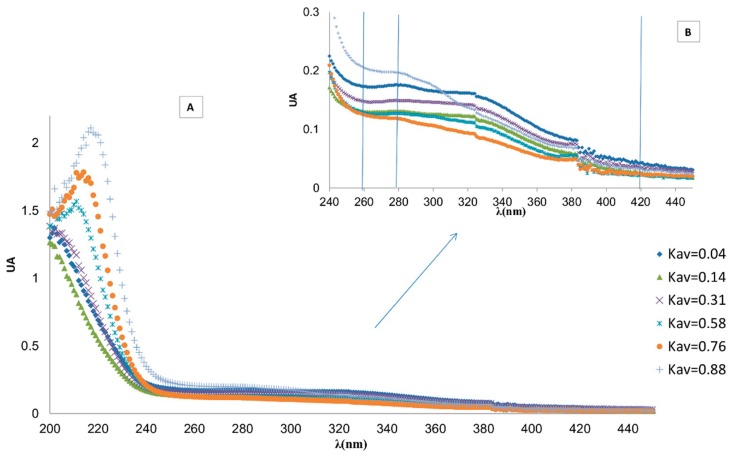
UV-VIS spectra of different melanoidins from IHS fractions isolated by SEC chromatography on a Biogel P2 column (**A**). Insert (**B**): expansion of the UV spectra.

The UV-VIS spectra of the chromatographic fractions of the soluble melanoidins of the Biogel-P2 column (Kav = 0.04 (Peak I), 0.14 (Peak II), 0.31, 0.58, 0.76, and 0.88 (Peak III)) are depicted in Figure 6. All fractions show a strong UV absorbance that extends to visible wavelengths, including those corresponding to yellow, orange, and brown [11,12,13,15].

As found previously for melanoidins isolated from beer [15], the UV spectra of the fractions showed differences in the maximum absorbance wavelength that point to structural variations.

**Figure 7:**
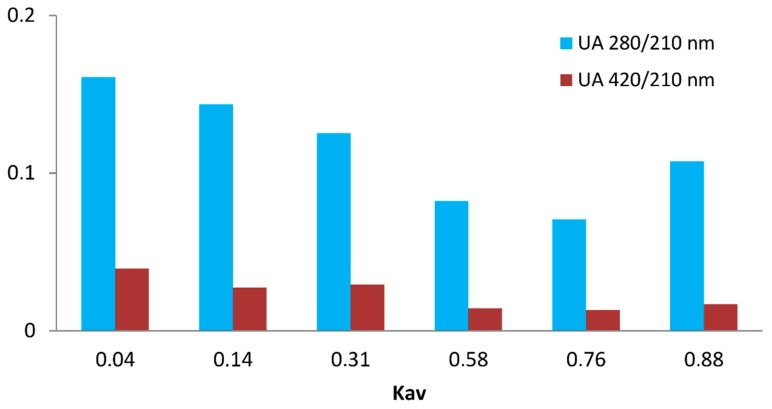
Bar diagram of the ratios of absorbances at λ 280 and λ 420 nm to absorbance at λ 210 nm for the fractions obtained by size-exclusion chromatography of IHS.

In Figure 7 the difference in the absorption spectra of the fraction is more clearly seen, where the ratios of absorbance at 280 and 420 nm to absorbance at 210 nm are represented (A_280_/A_210_ and A_420_/A_210_, respectively). Almost all organic chemical groups absorb UV light at 210 nm, so this value can be used as an approximation of concentration in each chromatographic fraction, regardless of the chemical nature of the compounds present in that fraction. Taking this into account, the ratios A280/A210 and A420/A210 can be considered as relative measures of the concentrations of aromatic compounds and colored compounds (melanoidins), respectively. The aromatic compounds should be expected to be mainly composed of aromatic amino acids (phenylalanine, tryptophan, tyrosine) [16] liberated from the action of pronase on the proteins present in the insoluble fraction, while the melanoidins were derived from products formed by the Maillard reaction. The higher molecular fractions of IHS showed aromatic character (higher A280/A210 ratio) and color (higher A420/A210 ratio) (Figure 7). The aromatic compounds eluting at Kav ~ 0.88 (peak III) may be composed of aromatic amino acids released by the hydrolysis of the melanoproteins.

Regarding the above data, the melanoidins fractions with Kav between 0.17 and 0.65 were selected for further structural characterization, corresponding to nominal MW from 400 up to1800 Da. The compounds eluting in peaks II and III were not considered to avoid the interference with amino acids or sugars.

### 2.6. Elemental Analysis of Melanoidins

The nitrogen (N) and carbon (C) contents of the melanoidins fraction and IHS were evaluated by elemental analysis, and the ratio N/C determined (Table 2).

The N/C ratio for the insoluble (I) and the melanoidins fractions from IHS are the same, and they are like those determined by Brands et al. [17] in a study about the incorporation of sugars to casein (N/C ratios from 0.23 to 0.25). Different N content values (6%) have been found in high MW melanoidins in model systems using glycine with glucose, fructose, or 5-(hydroxymethyl)-furfural (HMF) [18], and in glycine-glucose model systems [19].

The N/C ratio of the insoluble fraction (I) and the melanoidins fractions were similar, albeit the melanoidins showed a 0.6% sulfur content (Table 2) while the sulfur content in the insoluble fraction (I) was below the detection limit. The sulfur in melanoidins could come from cysteine, cystine, or thiazolidines generated by the Maillard reaction [19,20].

### 2.7. NMR Analysis of Melanoidins

The melanoidins fractions obtained by SEC from the hydrolysis products of the insoluble fraction of DL (IHS) consist of a complex mixture of products derived from different routes of the Maillard reaction. As a contribution to the preliminary characterization of these products, the fractions of soluble melanoidins from IHS were analyzed by nuclear magnetic resonance (NMR).

**Figure 8:**
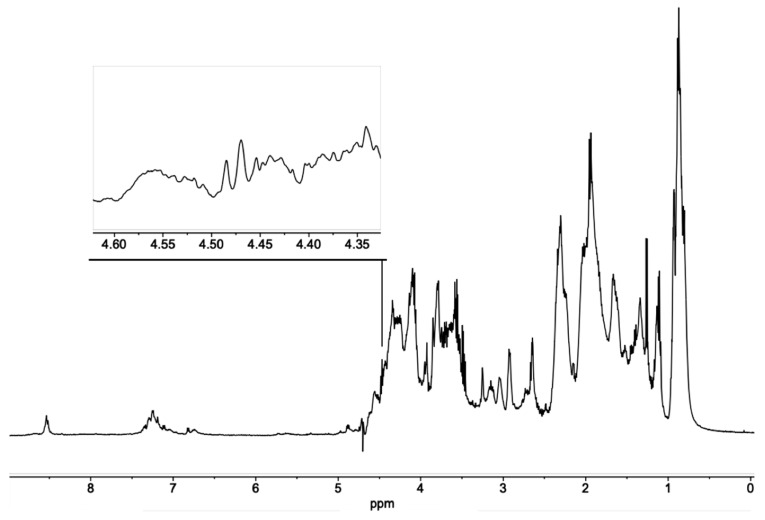
Water-suppressed ^1^H NMR spectrum of melanoidins from DL. The expansion shows the anomeric resonance of a β-D-galactopyranosyl residue at the non-reducing end of a lysine-linked lactose molecule.

**Figure 9:**
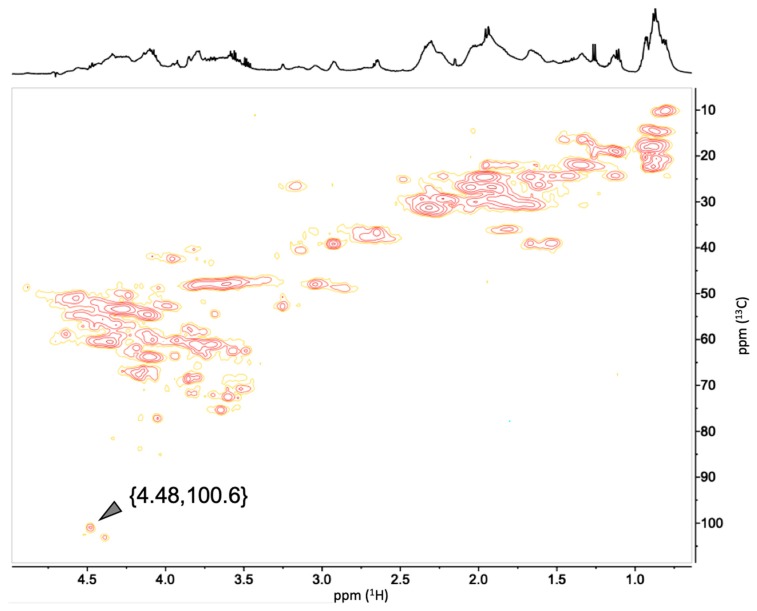
HSQC spectrum of melanoidins from DL melanoidins with nominal MW in the range 400 to 1800 Da.

The complexity of the melanoidins mixture is reflected in the ^1^H-NMR spectrum of the sample (Figure 8; Figure 9). The mixture could be composed of Amadori products from the initial steps of the Maillard reaction [21] up to colored products [8]. Due to the complexity of the ^1^H-NMR, no attempts were made to identify all the resonance signals, but instead, to use some of them to partially characterize chemical groups associated to potential products of the Maillard reaction. Of interest are resonances from aromatic protons (δ_H_ 8.5–7.0), those from protons linked to carbons with heteroatoms (δ_H_ 4.5–3.5), and from aliphatic protons (δ_H_ 2.5–0.5).

A correlation in the ^1^H,^13^C-hetero correlation spectrum (HSQC) of a proton resonance signal at δ_H_ 4.48 ppm (Figure 8) to a carbon signal at δ_C_ 100.6 ppm (Figure 8; Figure 9) can be assigned to the anomeric proton and carbon of a **β**-d-galactopyranosyl residue of a lactose molecule linked via its reducing end to an **ε**-amine group of a lysine residue [22]. This type of derivative is one of the initial Amadori products that can be formed in the initial chemical modifications that occur during the DL production, which is released in a soluble form only after the enzymatic hydrolysis of the melanoprotein with a nominal MW in the range 400 to 1800 Da. These products are expected to form during the complex sequence of reactions proposed to occur between the milk proteins and the sugars. One of the first transformations involves the condensation of the reducing end of lactose with the **ε**-amine group of a lysine residue from the protein via the formation of an imine, followed by the formation of the Amadori products and subsequent reactions. The galactose residue is preserved in the 1-deoxyosone and 3-deoxyosone routes since the related chemical modifications affect only the glucose residue at the reducing end of lactose. This mechanism agrees with the presence of the anomeric proton and carbon resonance signals mentioned above. Both hypotheses are in agreement with the experimental result, suggesting the initial formation of the Amadori product and its degradation via the formation of a 4-osazone with the simultaneous loss of the galactose residue [23,24].

### 2.8. Analysis of Melanoidins by HPLC-DAD

**Figure 10:**
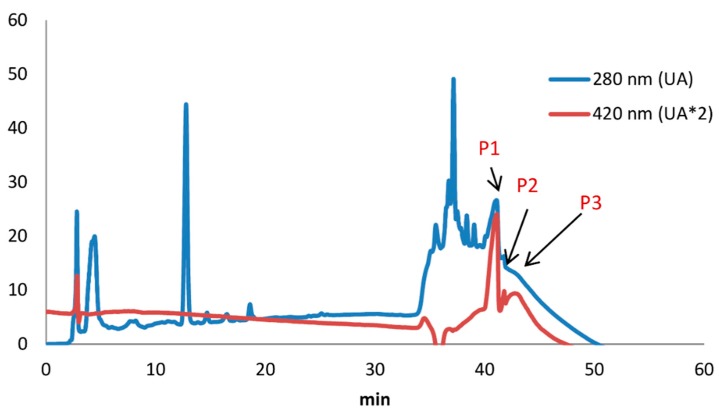
RP-HPLC-DAD chromatograms of melanoidins of nominal MW in the range 400 to 1800 Da, with detection at λ 280 and 420 nm.

Figure 10 shows the HPLC chromatograms of melanoidins eluted through a C18 column with detection at λ 280 nm and 420 nm. Even though the injected sample eluted in a rather wide MW range in gel-filtration, it showed a relatively simple HPLC-chromatographic profile. The chromatogram obtained at 280 nm showed peaks due to the presence of aromatic compounds, while the chromatogram at 420 nm indicates the presence of melanoidins. Both chromatograms show broad and non-completely resolved peaks, as observed in similar prior samples, with the suggestion that this may be ascribed to the presence of polymeric products which are entirely separated in this chromatographic system [25]. From the comparison of both chromatograms, more hydrophobic melanoidins present higher retention times compared with colorless aromatic compounds in general, which may be attributed to the higher degree of condensation and aromatization of the colored compounds produced in advances stages of the Maillard reaction.

## 3. Materials and Methods

### 3.1. DL Production

DL was elaborated in Granja Pocha S.A. using 60 L industrial kettle (IMAI, Santa Fe, Argentina) equipped with a vapor heating jacket and regulated mechanical stirring. Two production runs performed using milk with a protein content of 2.9–3.0% and fat content below 0.1%. The runs were made in the “full kettle” modality using 20 L milk added with sodium monostearate (0.02%) as anti-foaming. Commercial sucrose was added with continuous agitation at 60 °C to a total content of 20% relative to milk adjusting the initial pH to 7.50 ± 0.05 with sodium bicarbonate using a pH-meter equipped with a penetration electrode (Hanna HI8424, HANNA Instruments, RI, USA). The process led to a final concentration of solids of 67° Brix in approximately 2.5 h.

### 3.2. Isolation of the Insoluble Fraction from DL

Compounds with low MW, particularly sucrose, were removed by dialysis. For this purpose, DL (3 g) was dispersed in water (150 mL) using magnetic stirring (10 min). The resulting dispersion was dialyzed against water (3 × 2 L) using a membrane with a cut-off of 6–8 kDa (Spectra/Por, Spectrum Laboratories Inc., Rancho Dominguez, CA, USA) for 24 h.

The macromolecular components of DL were isolated following the procedure described by Le et al. [26], with some modifications. Namely, the dialyzed suspension of DL (40 mL) was centrifuged at 5500 rpm for 20 min at 25 °C, and the supernatant was collected and freeze-dried to give the soluble component (S). The precipitate was rinsed with water (2 × 15 mL), centrifuged, and freeze-dried to provide the insoluble macromolecular material (I). The soluble and insoluble proportion (%S and %I, respectively) of the macromolecular fraction of DL was calculated as the percent ratio of the mass of the S and I components relative to their sum. The average value was calculated from the results of three repetitions.

### 3.3. Enzymatic Hydrolysis of the Soluble and Insoluble Protein Fractions

The melanoidins were isolated from the macromolecular fractions obtained after dialysis following the protocol of Morales and van Boekel [11], but including enzymatic hydrolysis in the process. In a typical run, the I and S fractions were suspended in 50 mM phosphate buffer pH 7.0 (20 mg/mL) and treated with 1% Pronase E from *Streptomyces griseus* (4 UI/mg, Sigma-Aldrich, St. Louis, MI, USA) at 37 °C for 24 h with magnetic agitation, and then centrifuged at 4,500 rpm for 30 min at 4 °C. The supernatants obtained from the treatment of the I and S fractions were separated and labeled IHS and SH, respectively, and fractionated by SEC-HPLC on a Biogel P2 column irrigated with aqueous acetic acid (0.2% *v*/*v*). This process was repeated thrice.

### 3.4. SEC-HPLC of Melanoidins

The soluble protein fraction and the hydrolysis mixtures from the I and S fractions were analyzed by SEC-HPLC using a system consisting of a BioSep-SEC-S3000 column (Phenomenex, Torrance, CA, USA) and a UV-VIS detector (Shimadzu SPD-20A, Shimadzu, Kioto, Japan) at λ 280 nm. The column was irrigated with phosphate buffer (50 mM, pH 6.8), and calibrated using as Blue Dextran (2000 kDa), BSA (66 kDa), lysozyme (14.3 kDa), and phenylalanine (165 Da). This procedure was performed three times to asses repeatability.

### 3.5. Isolation of Melanoidins by Gel Filtration

The melanoidins released by hydrolysis of the Insoluble and Soluble protein fractions (IHS and SH, respectively) were isolated by gel filtration using a BioGel P2 (BioRad, Hercules, CA, USA) column (2.6 × 50 cm) irrigated with aqueous acetic acid (0.2% *v*/*v*) at a flow of 36 mL/h monitored by RI detection. Aliquots of 50 mg sample dissolved in 2 mL mobile phase were applied to the column, and fractions were collected every 5 min. The UV-VIS spectra (200–600 nm) spectrum of each fraction was recorded on a Pharmacia Ultrospec 1000 spectrophotometer (Pharmacia, Uppsala, Sweden). Similar fractions were pooled and freeze-dried. The procedure was repeated three times to produce enough material, and analogous fractions were pooled.

### 3.6. Elemental Analysis

The elemental analysis of the samples (2 mg each) was performed according to Hayashi and Namiki [27] using an elemental analyzer Carlo Erba EA1108 (Sabadell, Spain) to calculate the nitrogen/carbon ratio (N/C).

### 3.7. NMR Analysis

All NMR spectra were recorded at 25 °C on a Bruker AVANCE III 500 NMR spectrometer (Bruker Corp., Billerica, MA, USA) operating at ^1^H and ^13^C frequencies of 500.13 MHz and 125.76 MHz, respectively, and equipped with a *z*-gradient TXI probe. The samples were dissolved in D_2_O using sodium 2,2,3,3-*d*_4_-3-(trimethylsilyl)propionate (TSP) the internal standard. Water-suppressed ^1^H spectra were recorded using a 30-degree pulse and presaturation during a 2 s repetition delay, accumulating a total of 256 scans. HSQC spectra were obtained with a gradient-enhanced pulse sequence [28], using 128 scans per slice and a repetition delay of 1 s.

### 3.8. HPLC Analysis of Melanoidins

The melanoidins released by the hydrolysis of the melanoprotein I and S fractions were analyzed by HPLC following the method proposed by Bailey et al. [25]. The freeze-dried melanoidin fractions were weighed (6 mg) in a microcentrifuge tube, and methanol was added (1 mL). After stirring in an orbital shaker for 1 h, the mixture was centrifuged at 10,000 rpm for 20 min. The supernatant was collected, the solvent was evaporated under a nitrogen stream, and the residue was dissolved in aqueous methanol (5%, 200 µL). The solution was analyzed injecting 20 µL on a Dionex Ultimate 3000 HPLC (Thermo Scientific, Waltham, MA, USA), equipped with a C18 Zorbax Eclipse column (250 × 4.6 mm, 5 µm particle size) and a DAD detector, using a gradient of methanol in water from 5% to 35% in 30 min, followed by 100% methanol at 42 min at 1 mL/min flow.

## 4. Conclusions

The melanoidins of DL are mainly associated with a water-insoluble protein macromolecular fraction, in the form of high MW melanoproteins. The melanoidins released by enzymatic digestion of the melanoproteins are polydisperse with nominal MW ranging from 400 to 1800 Da, where the compounds with higher molecular weight contribute the most to the color as indicated by their absorbance at λ 420 nm. According to the UV-VIS and NMR spectra, the main components of the melanoidin fraction are aromatic and relatively more hydrophobic than other elements of the mixture resulting from the enzymatic treatment. Further studies of the compounds of the melanoidins isolated from the melanoproteins present in DL are necessary for the complete elucidation of their structure.

## Figures and Tables

**Table 1:** Composition of soluble (S) and insoluble (I) fractions in DL before.and after dialysis (α ≤ 0.05).

Sample	Component	% (Dry Weight)
DL before dialysis	Soluble	91.0 ± 3.0
	Insoluble	8.9 ± 3.0
DL after dialysis	Soluble (S)	70.0 ± 11.0
	Insoluble (I)	30.0 ±11.0
Insoluble hydrolyzed fraction (IH)	Soluble (IHS)	97.0 ± 2.0
	Insoluble (IHI)	3.3 ± 1.7

**Table 2:** Elemental composition of melanoidins fractions from IHS.

Sample	% N	% C	% H	% S	N/C Ratio
Melanoidins fraction	10.81	41.84	8.26	0.6	0.26
IHS	8.72	33.29	5.38	0.0	0.26
